# Integrative Genomic Analysis Predicts Regulatory Role of *N*^6^-Methyladenosine-Associated SNPs for Adiposity

**DOI:** 10.3389/fcell.2020.00551

**Published:** 2020-07-07

**Authors:** Weimin Lin, Hao Xu, Quan Yuan, Shiwen Zhang

**Affiliations:** ^1^State Key Laboratory of Oral Diseases, National Clinical Research Center for Oral Diseases, West China Hospital of Stomatology, Sichuan University, Chengdu, China; ^2^Department of Oral Implantology, West China Hospital of Stomatology, Sichuan University, Chengdu, China

**Keywords:** adiposity, genome-wide association study, m6A, single nucleotide polymorphism, epigenetics

## Abstract

Genome-wide association studies have identified many susceptible loci to explore the genetic factors of adiposity. However, the specific mechanisms by which these SNPs (single nucleotide polymorphism), particularly in the non-coding region, are involved in the pathogenesis of adiposity remain unclear. Recently, genetic variation is thought to affect *N*^6^-methyladenosine (m6A) RNA modification, which is the most common post-transcriptional messenger RNA modification. In this study, we identified a large number of BMI (body mass index)-associated m6A-SNPs from published GWAS summary statistics through a public database and explored their potential mechanisms involved in the pathogenesis of adiposity. In summary, the integrative analysis detected 20,993 BMI-associated m6A-SNPs and 230 m6A-SNPs which reached the genome-wide suggestive threshold (5.0E-05), while 215 of them showed eQTL signals and 167 are the corresponding genes. The leading SNP rs8024 (C/A) was located next to the m6A modification site of 3′UTR of the IPO9 gene, which was predicted to affect the m6A modification site and regulate the expression of the IPO9 gene to participate in the pathogenesis of adiposity. This m6A-SNP/gene expression/adiposity triplets provide a new annotation for the pathogenic mechanism of adiposity risk loci identified by GWAS.

## Introduction

Adiposity is considered to be both an independent disease and a clear risk factor that is closely related to the occurrence and death risk of non-communicable chronic diseases such as hypertension, cardiovascular and cerebrovascular disease, diabetes, and specific types of cancer, which has become one of the main sources of the burden of preventability worldwide (Apovian, 2016). Genome-wide association studies (GWAS) have identified a large number of potential risk loci and susceptible genes for exploring genetic factors of adiposity (Speakman et al., 2018). However, in addition to SNPs located in the coding regions of proteins which can directly affect amino acid sequences, many SNPs located in non-coding regions are also considered to affect epigenetic regulation and thus alter gene expression. FTO (fat mass and obesity associated gene) is the first gene associated with adiposity traits identified in the GWAS study (Frayling et al., 2007). Certain genetic variants of FTO gene appeared to be correlated with adiposity in human. Jia et al. (2011) revealed that *N*^6^-methyladenosine in nuclear RNA is a major substrate of FTO.

Similarly, our previous study also demonstrated that knockdown of m6A methylase METTL3 could promote the adipogenesis of bone marrow mesenchymal stem cells while inhibit osteogenesis, suggesting that m6A modification has an important role in adipogenesis and adiposity (Wu et al., 2018). m6A modification, first discovered in mammalian mRNAs in 1974, is considered to be the most abundant internal modification in eukaryotic mRNAs, and has been proven to be highly conserved in viruses, bacteria, yeast, plants, and vertebrates (Desrosiers et al., 1974; Perry and Kelley, 1974; Krug et al., 1976). m6A sites are mainly distributed in the stop codon regions, 3’UTR, 5’UTR and internal long exons of mRNA (Narayan et al., 1994). In recent years, the establishment of a sequencing method based on m6A antibody specific binding immunoprecipitation (MeRIP-Seq) proved that m6A modification was dynamically reversible, and made it possible to identify m6A modification sites across the whole-transcriptome (Dominissini et al., 2012; Meyer et al., 2012).

m6A modification could alter the structure, alternative splicing, stability, and translation of mRNA, thereby regulates gene expression, and is widely involved in the regulation of stem cell pluripotency, cell differentiation, neural maturation, and embryo development (Fu et al., 2014; Roundtree et al., 2017; Zhao et al., 2017; Frye et al., 2018). Some genetic variations in specific regions may affect m6A methylation and subsequent biological processes by changing the RNA sequence of target sites or key flanking nucleotides. These genetic variations are known as m6A-SNP (Zheng et al., 2018; Lin et al., 2019). Recently, Xiao et al. found an association between m6A modification and gene expression homeostasis in the whole-transcriptome m6A methylation across 21 major fetal tissues, revealing that human SNP sites with eQTL effects were enriched near the m6A modified region. It was confirmed that the SNP near the m6A modification site could affect the m6A modification of RNA (Xiao et al., 2019).

Therefore, m6A-SNP can be regarded as an important genetic functional variant, which can provide new clues for understanding the pathogenic molecular mechanisms of genetic variants. Till now, there are still no reports about the role of m6A-SNP in adiposity, and identification of m6A-SNP associated with adiposity could enrich the understanding of its genetic mechanism and the role of m6A modification in adiposity. Therefore, here we will use the public GWAS dataset to screen and explore the potential impact of m6A-SNP on adiposity.

## Materials and Methods

### Identify BMI-Associated m6A-Single Nucleotide Polymorphisms

By analysis of the BMI GWAS summary statistics with the m6A-SNP list, we identified the intersection of both, namely BMI-associated m6A-SNPs. Among them, BMI GWAS summary statistics is available on the GIANT website^1^, including a meta-analysis of previous GWAS of the GIANT consortium and GWAS of participants in UKBB, with a total sample size of approximately 0.7 million. The m6A-SNP list was downloaded from the m6Avar database^2^. The m6AVar database currently contained a high confidence of human m6A-SNP of 13,703 (miCLIP/PA-m6A-seq experiment), 54,222 medium (MeRIP-Seq experiment) and 245,076 (random genome prediction based on random forest algorithm) of low confidence. The 5.0E-05 was set as the suggestive threshold. In addition, we perform GO enrichment to explore the biological functions of these m6A-SNPs with an online website^3^ (Zhou et al., 2019).

### Functional Annotation of BMI-Associated m6A-SNPs

For the BMI-associated m6A-SNPs that have been identified, we used online tools to annotate them to further explore the mechanism by which they may play biological functions. The influence on the modification of nearby m6A site was obtained through m6AVar database query. One of the important potential pathways for these m6A-SNPs to play a biological role is affecting the post-transcriptional regulation of local genes. We have checked whether they have *cis*-eQTL effects in several different tissues and cells using the HaploReg browser (Ward and Kellis, 2016). In addition, we also queried about their other potential functions in transcription regulation, such as altering the binding capacity of transcription factors.

### Prediction of m6A Modifications Near m6A-SNP

Sequence-based RNA adenosine methylation site predictor (SRAMP) is a powerful m6A modification prediction tool based on a random forest classifier using genomic or cDNA sequences as input, which can predict the confidence thresholds of m6A modifications at different loci^4^ (Zhou et al., 2016). In order to determine the possibility of the identified m6A-SNPs affecting RNA modification, we used the online m6A modification prediction tool SRAMP with gene sequences of different genotypes as input to predict the changes of m6A modification.

### Differential Gene Expression Analysis

We further queried three transcriptome-wide studies on adiposity in the GEO public database. To verify whether m6A-SNPs showing *cis*-eQTL signals could affect local gene expression in the pathogenesis of adiposity. GSE88837 includes the gene expression level of visceral adipose tissues extracted during abdominal surgeries on 30 lean and obese adolescent females. GSE109597 includes whole peripheral blood samples from 90 healthy controls and overweight participants. GSE70353 includes a large-scale population study of subcutaneous adipose tissue gene expression level in 770 men with different BMI values. BMI is a person’s weight in kilograms divided by the square of height in meters. A high BMI can be an indicator of high body fatness, though there are slightly differences in BMI criteria for different genders and ages. Usually, the adults are regarded as overweight if their BMI is between 25 and 30 kg/m^2^, and obese when it is greater than 30 kg/m^2^ (Kuczmarski and Flegal, 2000). We used GEO2R online tool to calculate differentially expressed genes in adiposity (BMI > 30) and healthy (18.5 < BMI < 25) populations, and examined whether the BMI-associated genes (m6A-SNP with *cis*-eQTL) are differentially expressed between cases and controls. A significance level of *p* < 0.05 was used for differential expression analysis.

## Results

### Identification of BMI-Associated m6A-SNPs

**FIGURE 1:**
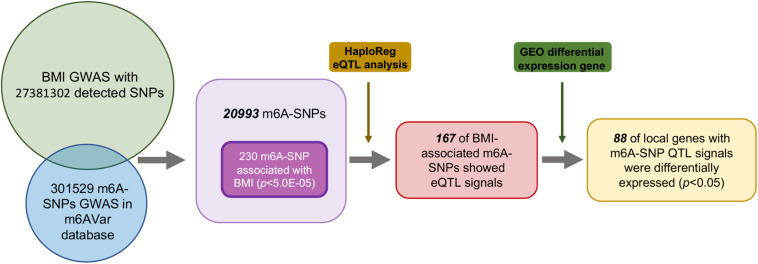
Flow chart of study design and the main results.

**FIGURE 2:**
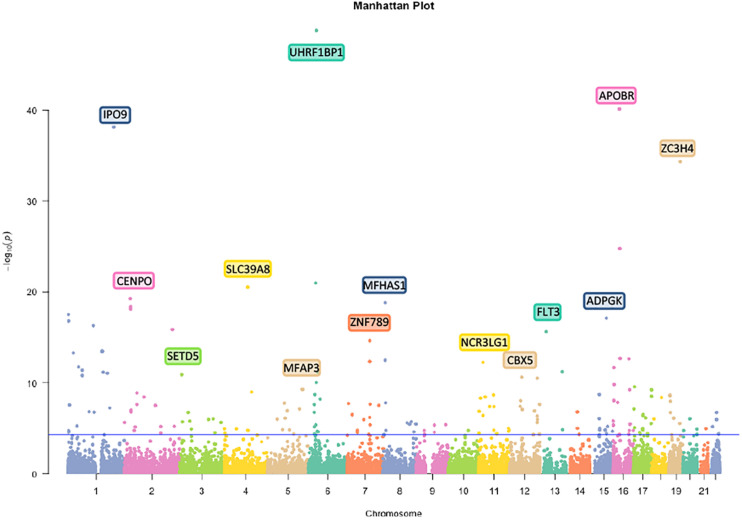
Manhattan plot of genome-wide identified BMI-associated m6A-SNPs. The Manhattan plot showed –log10(p.value) for each of 20993 m6A-SNPs associated with BMI. SNPs with association p.value less than 5.0E-05 was set as a suggestive threshold.

First, we analyzed 27381302 SNPs in BMI GWAS and 301529 m6A SNPs in the m6Avar database and identified 18568 m6A-SNPs on mRNA and 2425 m6A-SNPs related to m6A modifications on other RNAs (Figures 1, 2). These SNPs were not only located in the protein-coding gene regions, but also related to other RNA such as lincRNA, miRNA, and snoRNA. A large proportion of m6A-SNPs are distributed in exon regions (66%), including 54% in protein coding sequence and 12% in other exon regions. In addition, 29% of m6A-SNPs are distributed in 3′UTR, 5% of m6A-SNPs are distributed in 5′UTR, and a very small proportion of m6A-SNPs are distributed in the intron region (less than 1%) (Supplementary Figure S3). For the 20993 unique m6A-SNPs found in the GWAS dataset, 713, 3559, and 16721 m6A-SNPs were belonged to the high, medium, and low confidence region categories, respectively. Among them, the high-confidence m6A-SNP included variants that disrupt m6A motif obtained from miCLIP/PA-seq. For the medium-confidence m6A-SNP, variants that changed the sequence features for m6A modification in MeRIP-seq were included. The low-confidence m6A-SNP included variants near the genome-wide prediction based on random forest algorithm of m6A sequences. We used 5.0E-05 as the suggestive threshold to screen the association between these 20,993 m6A-SNPs and adiposity. Of these SNPs, 230 appeared to be associated with adiposity (*p* < 5.0E-05) (Supplementary Table S1). Through GO enrichment analysis, we found that the gene functions of these m6A-SNPs were enriched in biological processes related to transcriptional regulation, such as transcription factor binding, histone binding and methyltransferase activity, suggesting that these genes may affect transcriptional regulation (Supplementary Figure S1).

### Functional Annotation of BMI-Associated m6A-SNPs

To further explore the potential functional mechanisms of the 230 m6A-SNPs associated with adiposity, we investigated whether they were related to local gene expression level. In total, 215 BMI-associated m6A-SNPs tested (*p* < 0.05) showed *cis*-eQTL signals with 167 corresponding genes (Supplementary Table S1). Interestingly, by retrieving these m6A-SNPs in genotype-tissue expression (GTEx) database (Battle et al., 2017), we found that m6A-SNPs cause loss-of-function of m6A are more inclined to reduce local gene expression, and those gain-of-functions seem to have the reversed eQTL effect (Supplementary Figure S3). However, the mechanism of m6A modification affecting gene expression is very complicated, the impact of loss/gain of function of the m6A-SNPs on local gene expression is still remained to be explored. At the same time, to explore other possible mechanisms of these SNPs affecting transcriptional regulation, we queried the Haploreg browser and found that 20 of them changed the binding of one or more proteins in different cell types from the ENCODE transcription factor ChIP-seq dataset, and some of these transcriptional factors have been proved to be related with adiposity. For example, rs1802036 is related to the binding of transcriptional factor GATA1, which is considered to be a specific gene of brown adipose tissue (BAT). The increased expression of GATA1 is related to BAT formation, which may affect fat accumulation and lead to adiposity (Lu et al., 2012), suggesting that these SNPs may act as multifunctional variants to affect the transcriptional regulation of local genes (Table 1 and Supplementary Table S1).

**TABLE 1:** List of identified BMI-associated m6A-single-nucleotide polymorphisms.

Variant	Chr	Position	*P*.value	m6A_ID	Gene	Confidence_Level	m6A_ Function	Proteins bound	Motifs changed	eQTL	Mutation type
rs8024	1	201845575	7.10E-39	m6A_ID_1153	IPO9	miCLIP:(high)	Functional loss	Yes	No	10 hits	3′-UTR
rs4077410	16	29998200	1.71E-25	m6A_ID_42399	TAOK2	MeRIP-Seq:(medium)	Functional loss	No	Yes	73 hits	Synonymous
rs1046080	6	31595882	1.07E-21	m6A_ID_13969	PRRC2A	miCLIP:(high)	Functional loss	Yes	Yes	38 hits	Missense
rs9460	15	73044575	7.68E-18	m6A_ID_14394	ADPGK	miCLIP:(high)	Functional loss	No	Yes	1 hit	3′-UTR
rs10903311	8	8643387	3.26E-13	m6A_ID_77698	MFHAS1	MeRIP-Seq:(medium)	Functional loss	No	Yes	12 hits	3′-UTR
rs2255493	5	153432970	5.33E-10	m6A_ID_68479	MFAP3	MeRIP-Seq:(medium)	Functional loss	No	Yes	3 hits	Synonymous
rs1136948	16	3721773	8.66E-09	m6A_ID_42880	TRAP1	MeRIP-Seq:(medium)	Functional loss	No	Yes	46 hits	Missense
rs6953642	7	5540769	1.85E-08	m6A_ID_74738	FBXL18	MeRIP-Seq:(medium)	Functional loss	No	Yes	5 hits	Synonymous
rs2294532	1	6694927	2.72E-08	m6A_ID_43693	DNAJC11	MeRIP-Seq:(medium)	Functional loss	Yes	Yes	3 hits	3′-UTR
rs4233729	2	29092679	1.02E-07	m6A_ID_601471	TRMT61B	miCLIP:(high)	Functional loss	Yes	Yes	58 hits	Synonymous
rs2070489	3	38519424	1.82E-07	m6A_ID_64125	ACVR2B	MeRIP-Seq:(medium)	Functional loss	No	Yes	8 hits	Synonymous
rs3735440	7	21551070	3.16E-07	m6A_ID_74111	SP4	MeRIP-Seq:(medium)	Functional loss	No	Yes	4 hits	3′-UTR
rs7212573	17	42254281	3.23E-07	m6A_ID_46381	ASB16	MeRIP-Seq:(medium)	Functional loss	No	Yes	35 hits	Missense
rs5213	11	17408404	3.82E-07	m6A_ID_27449	KCNJ11	MeRIP-Seq:(medium)	Functional loss	Yes	Yes	16 hits	3′-UTR
rs1058587	19	18499422	7.53E-07	m6A_ID_700612	GDF15	PA-m6A-Seq:(high)	Functional loss	Yes	No	1 hit	Missense
rs3825175	12	122079441	2.25E-06	m6A_ID_32514	ORAI1	MeRIP-Seq:(medium)	Functional loss	No	Yes	5 hits	Synonymous
rs6859	19	45382034	2.86E-06	m6A_ID_6531	PVRL2	miCLIP:(high)	Functional loss	Yes	Yes	6 hits	3′-UTR
rs41264499	chr1	40090805	5.46E-06	m6A_ID_36999	HEYL	MeRIP-Seq:(medium)	Functional loss	No	Yes	2 hits	3′-UTR
rs3743481	chr15	77907145	6.29E-06	m6A_ID_40704	LINGO1	MeRIP-Seq:(medium)	Functional loss	No	Yes	1 hit	Synonymous
rs1049728	chr11	65421117	1.65E-05	m6A_ID_12688	RELA	miCLIP:(high)	Functional loss	Yes	Yes	5 hits	3′-UTR
rs2615542	chr4	104066461	1.68E-05	m6A_ID_65424	CENPE	MeRIP-Seq:(medium)	Functional loss	No	Yes	23 hits	Missense
rs260086	chr15	99674086	2.16E-05	m6A_ID_41227	SYNM	MeRIP-Seq:(medium)	Functional loss	No	No	5 hits	3′-UTR
rs8665	chr3	156259485	3.06E-05	m6A_ID_63457	SSR3	MeRIP-Seq:(medium)	Functional loss	No	No	10 hits	3′-UTR
rs12317704	chr12	132562291	4.41E-05	m6A_ID_32881	EP400	MeRIP-Seq:(medium)	Functional loss	No	Yes	25 hits	3′-UTR
rs12149862	chr16	69497203	4.55E-05	m6A_ID_43694	CYB5B	MeRIP-Seq:(medium)	Functional loss	No	Yes	7 hits	3′-UTR
rs4021	chr19	49253261	6.71E-05	m6A_ID_53257	FUT1	MeRIP-Seq:(medium)	Functional loss	No	Yes	9 hits	3′-UTR

**Only m6A-SNPs with medium or high confidence and differential local gene expression were selected*.*

### Differential Expression Analysis

For the above 167 m6A-SNPs showing *cis*-eQTL signals, we compared the expression level of local genes in three transcriptome studies of obese population. In these three datasets, we found that 88 of the 167 genes were differentially expressed in at least one study (*p* < 0.05) (Supplementary Table S1). For example, the established BMI-gene ADPGK expression is gradually downregulated in healthy, overweight, and adiposity populations (GSE109597, *p* = 0.00708315). We believe that m6A-SNP may be involved in the occurrence of adiposity by affecting the expression level of corresponding genes.

## Discussion

More than 100 chemical modifications have been detected on mRNA, of which *N*^6^-adenine (m6A) was one of the most extensive epigenetic modifications. During biological process such as cell differentiation, embryonic development, and stress, certain mRNAs can undergo modifications including *N*^1^-adenine methylation, *N*^5^-cytosine methylation, pseudouracil, and *N*^6^-adenine methylation (Boccaletto et al., 2018). Together, they form an apparently modified transcriptome for post-transcriptional regulation of mRNAs, enabling precise timing control of the process of mRNA translation into proteins, especially m6A modification that can regulate a series of biological processes in cells by regulating mRNA metabolism and translation.

Recent evidence suggested that m6A methylation could be involved in regulating blood glucose and lipid metabolism, and that abnormalities in m6A modification were increasingly linked to a variety of metabolic diseases (Peng et al., 2019; Wu et al., 2017; Zong et al., 2019). Nutritional challenges or dietary supplements have shown an effect on m6A methylation level, altering gene expression and phenotype. At the same time, some studies have demonstrated that the reduction of the overall level of m6A modification obtained by knocking down m6A methylase METTL3 could promote adipogenic differentiation (Yao et al., 2019). However, there is no report on the role of m6A-SNP in adiposity.

**FIGURE 3:**
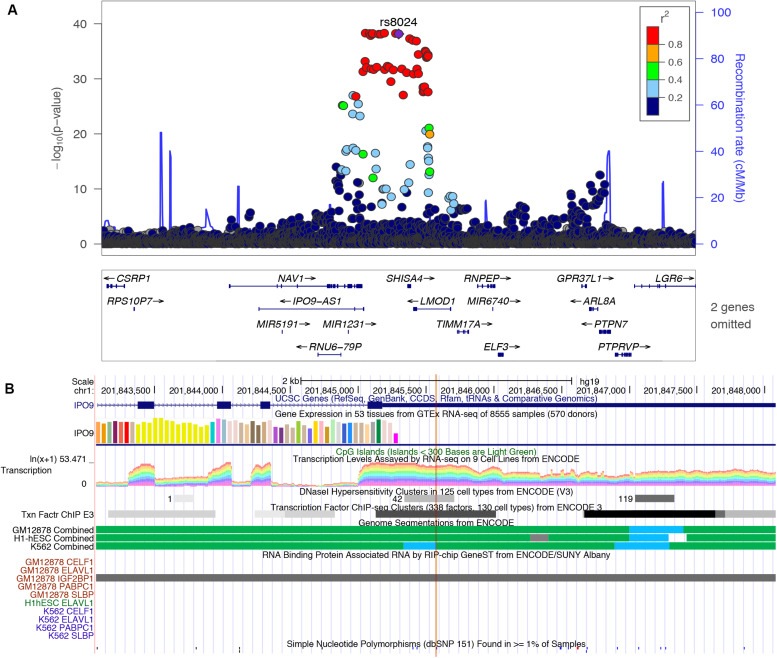
**(A)** Regional association plots of the rs8024 locus. The regional plot was plotted from published data of the BMI GWAS using the locus Zoom website tool (http://locuszoom.sph.umich.edu//). SNP rs8024 (purple) had a high degree of linkage disequilibrium (*r*^2^ > 0.8) with other high-risk SNP sites associated with BMI (*p* = 7.10E-39). **(B)** Integrative analysis of the potential function of SNP rs8024 in adiposity by querying USCS browser. rs8024 is located at the 3′UTR of IPO9. This region shows high transcription level and DNaseI hypersensitivity. RIP-chip GeneST from ENCODE/SUNY Albany data showed that the RNA-binding protein IGF2BP1 might have a potential interaction with rs8024-containing transcripts.

In this study, we found several m6A-SNP loci with high confidence that may be involved in adiposity. We used rs8024 located in the 3′UTR region of the IPO9 gene as an example for subsequent analysis. A recent study has shown that the IPO9 could interact with the JNK1/2 and promote their nuclear translocation (Zehorai and Seger, 2019). JNK1/2 has been proved to be an inhibitor of adipogenesis (Engin, 2017). Genetic variation in IPO9 may disrupt the nuclear localization of JNK1/2 and promote adipogenesis, thus leading to adiposity. rs8024 was located on the 3′UTR of the IPO9 gene exon on chromosome 1, and had a high degree of linkage disequilibrium (*r*^2^ > 0.8) with other high-risk SNP sites nearby (Figure 3). By querying the m6AVar database, we found that AGO2, EIF4A3, PTBP1, IGF2BP2, and IGF2BP3 were binding to the rs8024-related RNA region in IPO9 transcript in the CLIPdb and starBase2 databases. Among them, the IGF2BP family has recently been proven to be an m6A reader, which was believed to stabilize mRNA by binding to the m6A site on the mRNA. Knockdown of IGF2BP brought about a down-regulation of target gene expression (Huang et al., 2018). Therefore, rs8024 might lead to the loss of nearby m6A modifications, making the corresponding m6A reader unable to recognize this site and reducing gene expression. On the UCSC genome browser, we also found that the transcript of IPO9 had a binding site to the RNA-binding protein IGF2BP1 (Figure 3).

**FIGURE 4:**
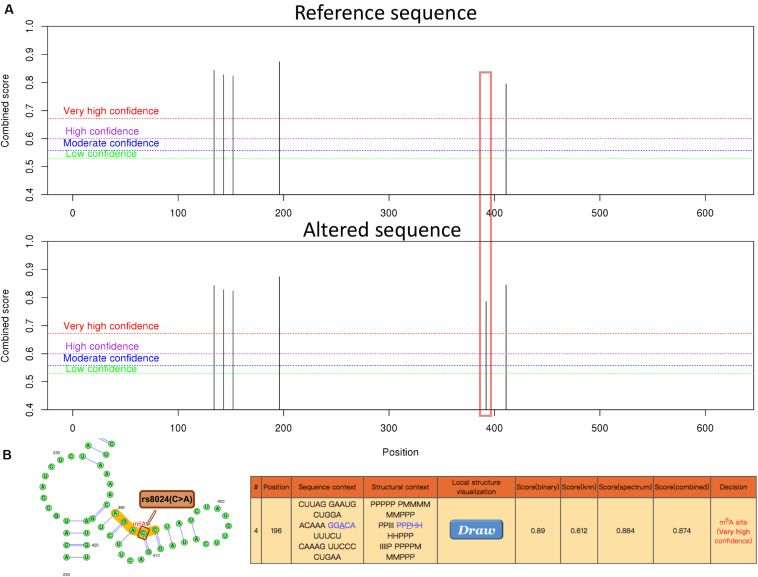
The genomic sequence of a representative IPO9 transcript (ENST00000361565.9) was used to predict the m6A modification on website (http://www.cuilab.cn/sramp), and the analysis of secondary structures was enabled. **(A)** An overview of the result page. The red box illustrates the disappearance of the m6A modification near rs8024 after the input of the altered sequence. **(B)** A graphical representation of the local secondary structure context around the mutation site rs8024. rs8024 (C > A) (brown) is very close to the predicted m6A modification site (red) with very high confidence. The H, M, I, B, and P in the secondary structure strings mean hairpin loop, multiple loop, interior loop, bulged loop, and paired residues, respectively.

In addition, possible m6A methylation sites IPO9 transcript sequence was predicted on the SRAMP website. After input of the IPO9 reference sequence, we found a highly convincible m6A modified predicted peak near rs8024, but this predicted peak disappeared after entering the altered sequence of IPO9, which also proved that rs8024 would affect m6A modification (Figure 4). To explore the mechanism in which rs8024 affects adiposity, we performed a phenotypic study on GWAS Atlas^5^ and found that rs8024 was also related to also related to daytime napping (*p* = 3.21E-20) and hypertension (*p* = 8.65E-12) (Supplementary Figure S2 and Supplementary Table S2).

**FIGURE 5:**
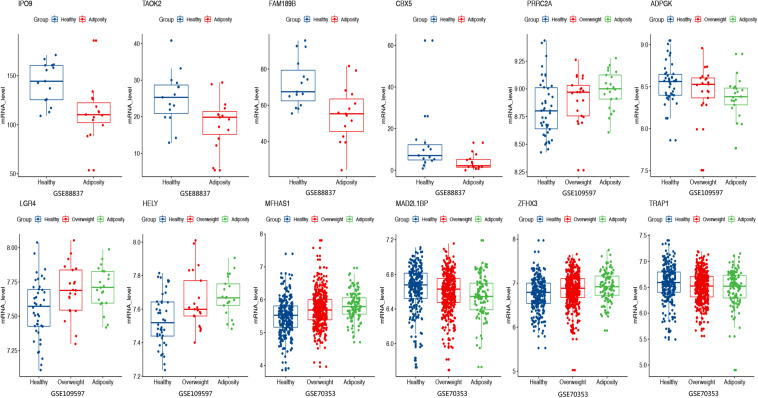
Expression levels of selected genes were displayed among adiposity and healthy populations in GSE88837 (visceral adipose tissue), GSE109597 (peripheral blood sample), and GSE70353 (subcutaneous adipose tissue).

Except for affecting m6A modification to change gene expression, m6A-SNP may also have other ways to participate in the regulation of gene expression. By querying the Haploreg database, we found that rs8024 was also located in the DNaseI hypersensitivity cluster region and would affect the binding of three transcription factors. Therefore, rs8024 should be considered a multifunctional variant involved in adiposity. As for expression level, it was found that mRNA expression of more than half of the m6A-SNP-associated genes was statistically different in at least one dataset (Figure 5 and Supplementary Table S1). In conclusion, we demonstrated that the identified m6A-SNPs may affect adiposity through giving rise to abnormal gene expression.

## Conclusion

In this study, we identified a total of 230 m6A-SNPs that may be associated with adiposity, and explored their potential functions using public databases. However, further research is still needed to verify how these m6A-SNPs affect m6A modification and its practical impact on the pathogenesis of adiposity. It is known that besides influencing global gene expression level, m6A-SNPs could also involve in disease progression by affecting the ratio between different RNA isoforms and the translation level of their protein products. However, such data are currently publicly limited, so only the potential impact of m6A-SNPs on gene expression level was explored in this study. Moreover, with the development of different detection m6A technologies, such as antibody-free m6A sequencing and nanopore sequencing, more accurate, disease-specific m6A sites would be identified. Genetic variants found near these sites have more possibilities to influence the pathogenesis of human diseases, so there are still more potential m6A-SNPs needed to be determined.

## Data Availability Statement

The datasets generated for this study can be found in the Data of BMI GWAS were publicly available at GIANT consortium (http://portals.broadinstitute.org/collaboration/giant/index.php/Main_Page). The m6ASNPs data can be downloaded from the m6AVar database (http://m6avar.renlab.org/). The three public gene expression data sets, GSE88837, GSE109597, and GSE70353 can be downloaded from GEO database (https://www.ncbi.nlm.nih.gov/geo/).

## Author Contributions

WL designed the study and wrote the manuscript. HX generated the figures and table. QY checked and polished the language. SZ designed the study and reviewed the manuscript.

## Conflict of Interest

The authors declare that the research was conducted in the absence of any commercial or financial relationships that could be construed as a potential conflict of interest.

## Footnotes

1 http://portals.broadinstitute.org/collaboration/giant/index.php/Main_Page

2 http://m6avar.renlab.org/

3 http://metascape.org/gp/index.html#/main/step1

4 http://www.cuilab.cn/sramp/

5 https://atlas.ctglab.nl/

## Supplementary Material

FIGURE S1 Up-keywords enrichment analysis was performed in candidate genes annotated to potential functional BMI-associated m6A-SNPs (*p* < 5.0E-05).

FIGURE S2 An overview of pheWAS study reveals the association between rs8024 and systemic phenotypes in GWAS Atlas.

FIGURE S3 **(A)** The effect of m6A-SNPs causing loss-of-function on gene expression. **(B)** The effect of m6A-SNPs causing gain-of-function on gene expression. **(C)** The distribution of adiposity-related m6A-SNPs in different gene regions.

TABLE S1 The identified 230 BMI-associated m6A-SNPs (*p* < 5.0E-05).

TABLE S2 List of the association between rs8024 and systemic phenotypes in GWAS Atlas.

## References

[B1] Apovian C. M. (2016). Obesity: definition, comorbidities, causes, and burden. *Am. J. Manag. Care* 22 s176–s185. 27356115

[B2] Battle A. Brown C. D. Engelhardt B. E. Montgomery S. B. (2017). Genetic effects on gene expression across human tissues. *Nature* 550 204–213. 10.1038/nature24277 29022597 PMC5776756

[B3] Boccaletto P. Machnicka M. A. Purta E. Piatkowski P. Baginski B. Wirecki T. K. (2018). MODOMICS: a database of RNA modification pathways. 2017 update. *Nucleic Acids Res.* 46 D303–D307. 29106616 10.1093/nar/gkx1030 PMC5753262

[B4] Desrosiers R. Friderici K. Rottman F. (1974). Identification of methylated nucleosides in messenger RNA from Novikoff hepatoma cells. *Proc. Natl. Acad. Sci. U.S.A.* 71 3971–3975. 10.1073/pnas.71.10.3971 4372599 PMC434308

[B5] Dominissini D. Moshitch-Moshkovitz S. Schwartz S. Salmon-Divon M. Ungar L. Osenberg S. (2012). et.al.Topology of the human and mouse m6A RNA methylomes revealed by m6A-seq. *Nature* 485 201–206. 10.1038/nature11112 22575960

[B6] Engin A. (2017). Human protein kinases and obesity. *Adv. Exp. Med. Biol.* 960 111–134. 10.1007/978-3-319-48382-5_5 28585197

[B7] Frayling T. M. Timpson N. J. Weedon M. N. Zeggini E. Freathy R. M. Lindgren C. M. (2007). A common variant in the FTO gene is associated with body mass index and predisposes to childhood and adult obesity. *Science* 316 889–894. 17434869 10.1126/science.1141634 PMC2646098

[B8] Frye M. Harada B. T. Behm M. He C. (2018). RNA modifications modulate gene expression during development. *Science* 361 1346–1349. 10.1126/science.aau1646 30262497 PMC6436390

[B9] Fu Y. Dominissini D. Rechavi G. He C. (2014). Gene expression regulation mediated through reversible m6A RNA methylation. *Nat. Rev. Genet.* 15 293–306. 10.1038/nrg3724 24662220

[B10] Huang H. Weng H. Sun W. Qin X. Shi H. Wu H. (2018). Recognition of RNA N(6)-methyladenosine by IGF2BP proteins enhances mRNA stability and translation. *Nat. Cell. Biol.* 20 285–295. 10.1038/s41556-018-0045-z 29476152 PMC5826585

[B11] Jia G. Fu Y. Zhao X. Dai Q. Zheng G. Yang Y. (2011). N6-methyladenosine in nuclear RNA is a major substrate of the obesity-associated FTO. *Nat. Chem. Biol.* 7 885–887. 10.1038/nchembio.687 22002720 PMC3218240

[B12] Krug R. M. Morgan M. A. Shatkin A. J. (1976). Influenza viral mRNA contains internal N6-methyladenosine and 5’-terminal 7-methylguanosine in cap structures. *J. Virol.* 20 45–53. 10.1128/jvi.20.1.45-53.1976 1086370 PMC354964

[B13] Kuczmarski R. J. Flegal K. M. (2000). Criteria for definition of overweight in transition: background and recommendations for the United States. *Am. J. Clin. Nutr.* 72 1074–1081. 10.1093/ajcn/72.5.1074 11063431

[B14] Lin W. Xu H. Wu Y. Wang J. Yuan Q. (2019). In silico genome-wide identification of m6A-associated SNPs as potential functional variants for periodontitis. *J. Cell. Physiol.* 235 900–908. 10.1002/jcp.29005 31245852

[B15] Lu X. Ji Y. Zhang L. Zhang Y. Zhang S. An Y. (2012). Resistance to obesity by repression of VEGF gene expression through induction of brown-like adipocyte differentiation. *Endocrinology* 153 3123–3132. 10.1210/en.2012-1151 22593269

[B16] Meyer K. D. Saletore Y. Zumbo P. Elemento O. Mason C. E. Jaffrey S. R. (2012). Comprehensive analysis of mRNA methylation reveals enrichment in 3’ UTRs and near stop codons. *Cell* 149 1635–1646. 10.1016/j.cell.2012.05.003 22608085 PMC3383396

[B17] Narayan P. Ludwiczak R. L. Goodwin E. C. Rottman F. M. (1994). Context effects on N6-adenosine methylation sites in prolactin mRNA. *Nucleic Acids Res.* 22 419–426. 10.1093/nar/22.3.419 8127679 PMC523598

[B18] Peng S. Xiao W. Ju D. Sun B. Hou N. Liu Q. (2019). Identification of entacapone as a chemical inhibitor of FTO mediating metabolic regulation through FOXO1. *Sci. Transl. Med.* 11:eaau7116. 10.1126/scitranslmed.aau7116 30996080

[B19] Perry R. P. Kelley D. E. (1974). Existence of methylated messenger RNA in mouse L cells. *Cell* 1 37–42. 10.1016/0092-8674(74)90153-6

[B20] Roundtree I. A. Evans M. E. Pan T. He C. (2017). Dynamic RNA modifications in gene expression regulation. *Cell* 169 1187–1200. 10.1016/j.cell.2017.05.045 28622506 PMC5657247

[B21] Speakman J. R. Loos R. J. F. O’Rahilly S. Hirschhorn J. N. Allison D. B. (2018). GWAS for BMI: a treasure trove of fundamental insights into the genetic basis of obesity. *Int. J. Obes.* 42 1524–1531. 10.1038/s41366-018-0147-5 29980761 PMC6115287

[B22] Ward L. D. Kellis M. (2016). HaploReg v4: systematic mining of putative causal variants, cell types, regulators and target genes for human complex traits and disease. *Nucleic Acids Res.* 44 D877–D881. 26657631 10.1093/nar/gkv1340 PMC4702929

[B23] Wu W. Feng J. Jiang D. Zhou X. Jiang Q. Cai M. (2017). AMPK regulates lipid accumulation in skeletal muscle cells through FTO-dependent demethylation of N(6)-methyladenosine. *Sci. Rep.* 7:41606. 10.1038/srep41606 PMC5296945 28176824

[B24] Wu Y. Xie L. Wang M. Xiong Q. Guo Y. Liang Y. (2018). Mettl3-mediated m(6)A RNA methylation regulates the fate of bone marrow mesenchymal stem cells and osteoporosis. *Nat. Commun.* 9:4772. 10.1038/s41467-018-06898-4 PMC6235890 30429466

[B25] Xiao S. Cao S. Huang Q. Xia L. Deng M. Yang M. (2019). The RNA N(6)-methyladenosine modification landscape of human fetal tissues. *Nat. Cell. Biol.* 21 651–661. 10.1038/s41556-019-0315-4 31036937

[B26] Yao Y. Bi Z. Wu R. Zhao Y. Liu Y. Liu Q. (2019). METTL3 inhibits BMSC adipogenic differentiation by targeting the JAK1/STAT5/C/EBPbeta pathway via an m(6)A-YTHDF2-dependent manner. *FASEB J.* 33 7529–7544. 10.1096/fj.201802644r 30865855

[B27] Zehorai E. Seger R. (2019). Beta-like importins mediate the nuclear translocation of MAPKs. *Cell. Physiol. Biochem.* 52 802–821. 10.33594/000000056 30946556

[B28] Zhao B. S. Roundtree I. A. He C. (2017). Post-transcriptional gene regulation by mRNA modifications. *Nat. Rev. Mol. Cell Biol.* 18 31–42. 10.1038/nrm.2016.132 27808276 PMC5167638

[B29] Zheng Y. Nie P. Peng D. He Z. Liu M. Xie Y. (2018). m6AVar: a database of functional variants involved in m6A modification. *Nucleic Acids Res.* 46 D139–D145. 29036329 10.1093/nar/gkx895 PMC5753261

[B30] Zhou Y. Zeng P. Li Y.-H. Zhang Z. Cui Q. (2016). SRAMP: prediction of mammalian N6-methyladenosine (m6A) sites based on sequence-derived features. *Nucleic Acids Res.* 44:e91. 10.1093/nar/gkw104 26896799 PMC4889921

[B31] Zhou Y. Zhou B. Pache L. Chang M. Khodabakhshi A. H. Tanaseichuk O. (2019). Metascape provides a biologist-oriented resource for the analysis of systems-level datasets. *Nat. Commun.* 10: 1523. 10.1038/s41467-019-09234-6 PMC6447622 30944313

[B32] Zong X. Zhao J. Wang H. Lu Z. Wang F. Du H. (2019). Mettl3 deficiency sustains long-chain fatty acid absorption through suppressing Traf6-dependent inflammation response. *J. Immunol.* 202 567–578. 10.4049/jimmunol.1801151 30567729 PMC6321842

