# Supplementary figures and images for: Integrative Genomic Analysis Predicts Regulatory Role of N6-Methyladenosine-Associated SNPs for Adiposity
Source: Front Cell Dev Biol. 2020 Jul 7;8:551. doi: 10.3389/fcell.2020.00551 (PMC7358408; doi:10.3389/fcell.2020.00551)

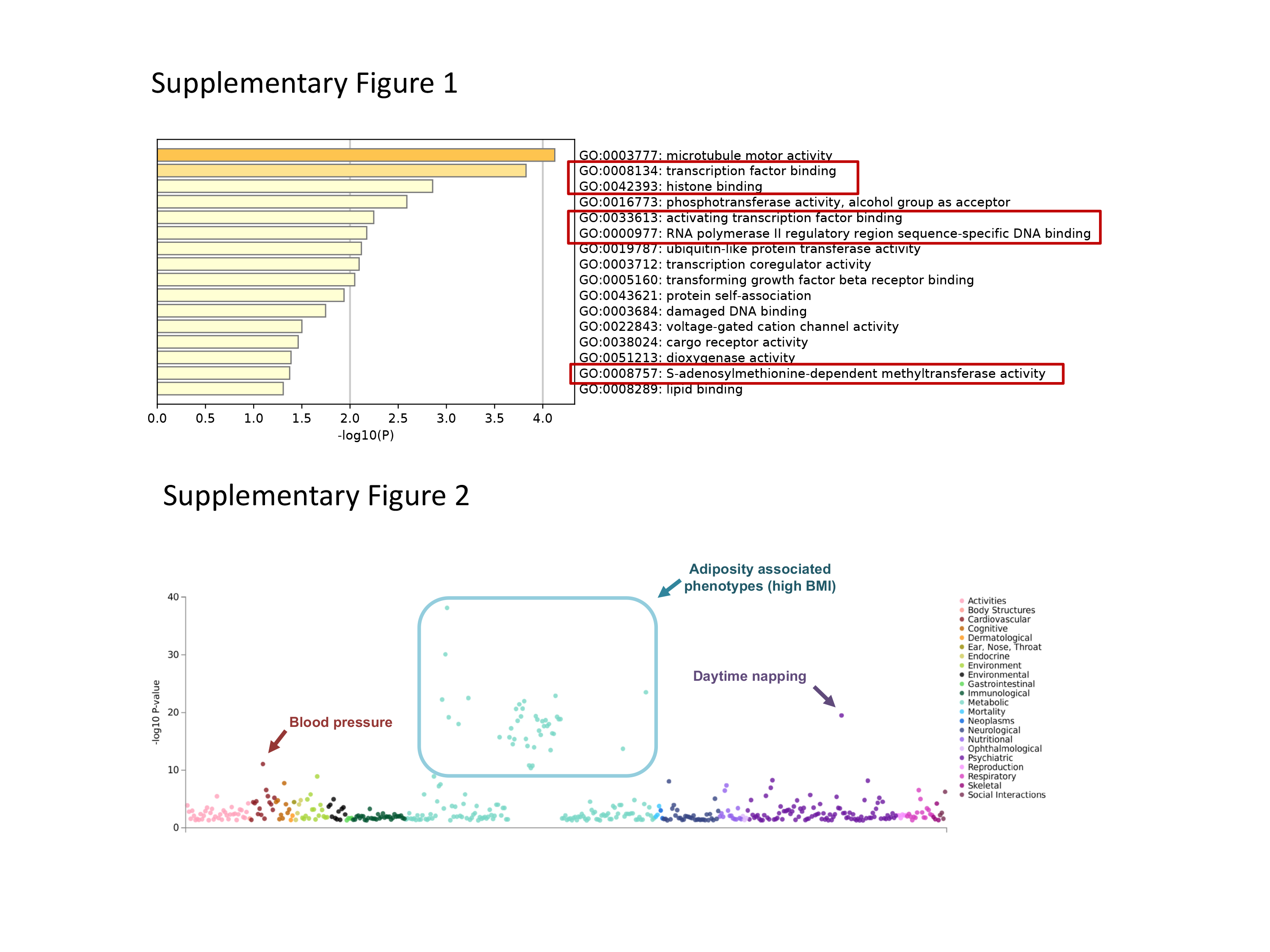

Supplement: FIGURE S1 — Up-keywords enrichment analysis was performed in candidate genes annotated to potential functional BMI-associated m6A-SNPs (p < 5.0E-05). [file Image_1.TIF]

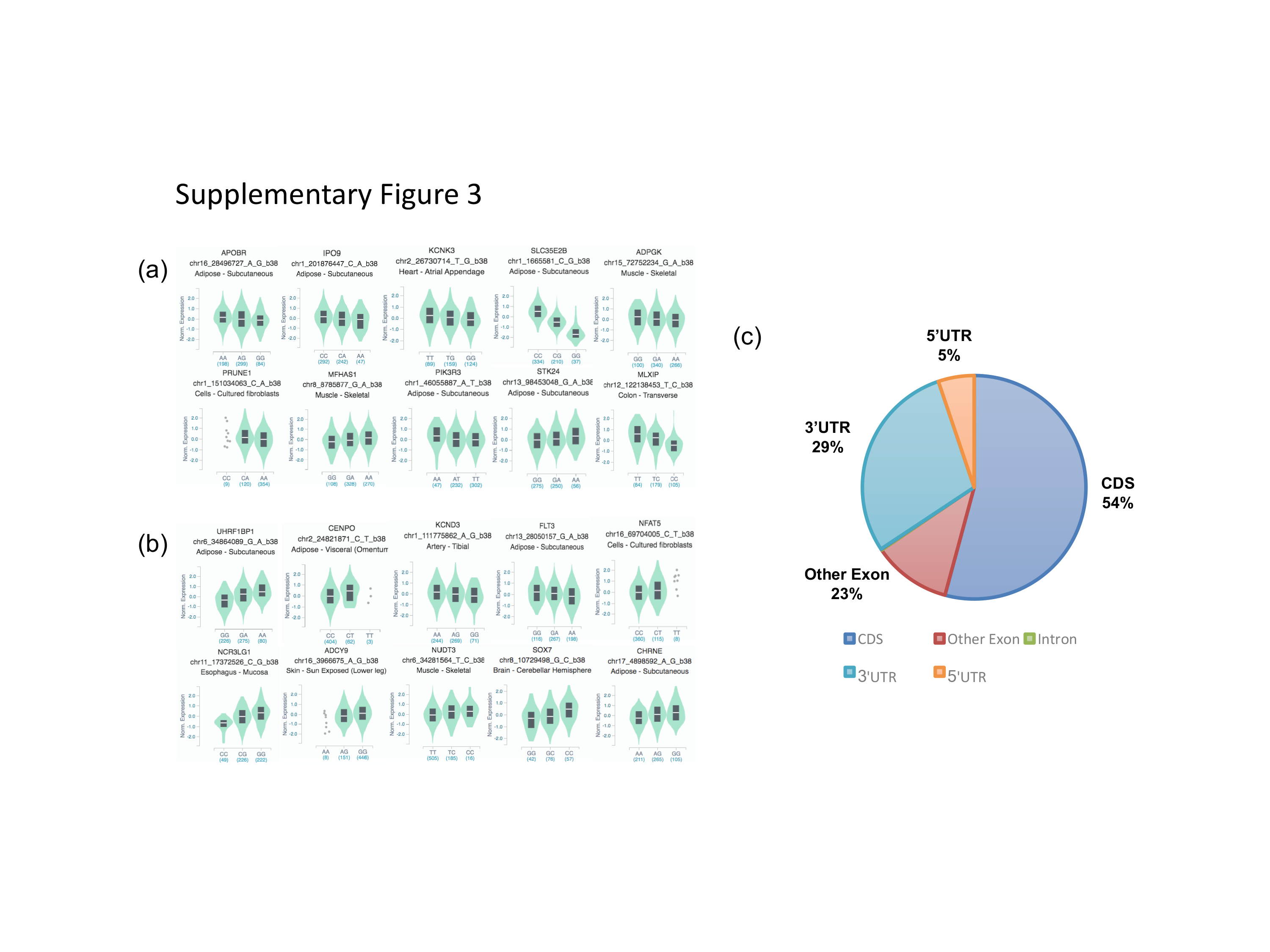

Supplement: FIGURE S3 — (A) The effect of m6A-SNPs causing loss-of-function on gene expression. (B) The effect of m6A-SNPs causing gain-of-function on gene expression. (C) The distribution of adiposity-related m6A-SNPs in different gene regions. [file Image_2.TIF]
